# Denervation‐induced muscle atrophy suppression in renalase‐deficient mice via increased protein synthesis

**DOI:** 10.14814/phy2.14475

**Published:** 2020-08-01

**Authors:** Katsuyuki Tokinoya, Takanaga Shirai, Yuya Ota, Tohru Takemasa, Kazuhiro Takekoshi

**Affiliations:** ^1^ Doctoral Program in Sports Medicine Graduate School of Comprehensive Human Sciences University of Tsukuba Tsukuba Japan; ^2^ Research Fellow of the Japan Society for the Promotion of Science Tokyo Japan; ^3^ Graduate School of Comprehensive Human Sciences University of Tsukuba Tsukuba Japan; ^4^ Faculty of Health and Sport Sciences University of Tsukuba Tsukuba Japan; ^5^ Division of Clinical Medicine Faculty of Medicine University of Tsukuba Tsukuba Japan

**Keywords:** Akt, mitochondria, p70S6K, skeletal muscle

## Abstract

Denervation‐induced muscle atrophy increases signaling through both protein degradation and synthesis pathways. Renalase is a flavin adenine dinucleotide‐dependent amine oxidase that inhibits apoptosis and inflammation and promotes cell survival. This study aimed to elucidate the effect of renalase on denervation‐induced muscle atrophy. We used 7‐week‐old renalase knock‐out (KO) mice (a model of denervation‐induced muscle atrophy) and wild‐type (WT) mice (KO: *n* = 6, weight = 20–26 g; WT: *n* = 5, weight = 19–23 g). After their left legs were denervated, these mice were killed 1 week later. KO mice had lighter muscle weight than the WT mice. We observed an increase in molecular signaling through protein degradation pathway as well as oxidative stress in denervated muscles compared with that in sham‐operated muscles in both WT and KO mice. Additionally, we also observed the main effect of renalase in WT and KO mice. Mitochondrial oxidative phosphorylation protein content was lower in denervated muscles than in sham‐operated muscles in both WT and KO mice. However, a significant difference was noted in the reaction with Akt and p70S6K (components of the protein synthesis pathway) between WT and KO mice. In conclusion, mice with renalase deficiency demonstrated an attenuation of denervation‐induced muscle atrophy. This might be related to catecholamines because signaling through the protein synthesis pathway was increased following denervation in renalase KO mice compared with that in WT mice, despite showing no change in signaling through protein degradation pathways.

## Graphical abstract

We show that signaling through the protein synthesis pathway in the denervated limb of renalase knock‐out mice was increased compared with wild‐type mice, despite no change in signaling through protein degradation pathways.

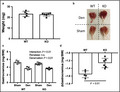

## 1 INTRODUCTION

Skeletal muscle comprises approximately 40% of body mass in humans. Muscle is a plastic tissue that can be hypertrophic or atrophic. Muscle atrophy may be caused by aging, malnutrition, and physical inactivity. In rodent models, muscle atrophy can be induced by cutting the sciatic nerve (denervation), thereby immobilizing the skeletal muscle (Argadine, Mantilla, Zhan, & Sieck, 2010; DePinho et al., 2014). Muscle protein degradation may occur from increased activity through various protein degradation pathways, including the ubiquitin–proteasome pathway, autophagy, and caspase (Braun et al., 2017). In contrast, denervation‐induced muscle atrophy is associated with an increased phosphorylation of components of the protein synthesis pathway (e.g. Akt, mTOR and S6K; Argadine et al., 2010; DePinho et al., 2014; Machida et al., 2011). Machida et al. (2011) reported that the ratio of phosphorylated/total p70S6K (a marker of mTOR activity) was increased by 397% in denervated muscles compared with that in sham‐operated muscles. However, denervation is one of several different muscle atrophy models, and it does not necessarily follow that other models are also associated with increased protein synthesis.

Renalase is a flavin adenine dinucleotide‐dependent amine oxidase (Xu et al., 2005), and its primary function is to metabolize catecholamines and regulate blood pressure (Wu et al., 2011; Xu et al., 2005). Previous studies have suggested that renalase also has an inhibitory effect on apoptosis and inflammation, thereby promoting cell survival (Huang et al., 2019; Wu et al., 2018; Yin et al., 2016; Zhao et al., 2015). Renalase exerts its protective effect by phosphorylating Akt via the renalase receptor plasma membrane calcium ATPase isoform 4b (PMCA 4b; Wang, Velazquez, Chang, Safirstein, & Desir, 2015; Wang et al., 2014). One study showed that in exercising skeletal muscle, renalase protects the cell via the ubiquitin–proteasome pathway by decreasing phosphorylation of protein kinase B (Akt; Tokinoya et al., 2018). On the other hand, a subsequent study from the same group suggests that this effect may result from renalase metabolizing catecholamines during exercise (Tokinoya, Yoshida, Sugasawa, & Takekoshi, 2020; Yoshida, Sugasawa, Tokinoya, Namba, & Takekoshi, 2017). Previous studies of renalase during exercise reported that renalase expression in the skeletal muscle increased with acute exercise despite a decrease in its expression in the kidney (Tokinoya et al., 2018; 2020). In addition, Tokinoya et al. (2020) showed that renalase concentration in blood increased after exercise. Thus, it is possible that renalase in the skeletal muscle plays a role during exercise. Additionally, renalase might metabolize adrenaline in skeletal muscle, as the administration of adrenaline to cultured myotubes increased renalase mRNA expression (Yoshida et al., 2017). Therefore, renalase plays two roles within the skeletal muscle: cell protection and catecholamine metabolism.

Renalase knock‐out (KO) mice have increased circulating concentrations of catecholamines (Quelhas‐Santos et al., 2015; Wu et al., 2011). Adrenaline has α‐ and β‐ receptors in tissues. Clenbuterol (a β2‐adrenal receptor agonist) attenuates muscle atrophy (Herrera, Zimmerman, Dykstra, & Thompson, 2001; Umeki et al., 2015) and phosphorylates Akt (Umeki et al., 2015). Within skeletal muscle, phosphorylated Akt stimulates protein synthesis and degradation and leads to muscle hypertrophy. Also, stimulation of cardiac β‐adrenal receptors resulted in phosphorylation of p70S6K in cardiomyocytes (Simm, Schlüter, Diez, Piper, & Hoppe, 1998). Thus, catecholamines could reduce muscle atrophy via the β2‐adrenal receptor in skeletal muscle.

This study aimed to elucidate the influence of renalase deficiency on denervation‐induced muscle atrophy by examining the effects on muscle protein synthesis and degradation pathways. We hypothesized, firstly, that skeletal muscle atrophy would be attenuated via increased signaling through the protein synthesis pathway, since renalase deficiency is associated with increased circulating catecholamines. We further hypothesized that skeletal muscle atrophy would be promoted via increased signaling through protein degradation pathways, since renalase deficiency would result in decreased cell protection via phosphorylation of Akt.

## 2 MATERIALS AND METHODS

### 2.1 Animals and experimental design

This study was approved by the Animal Subjects Committee, University of Tsukuba, Japan (approval number: 18‐425). B6;129S1‐Rnls^tm1Gvd^/J mice were purchased from Jackson Laboratory. Homozygotes were produced by mating heterozygous mice. Renalase KO and wild‐type (WT) mice were identified by PCR using genomic DNA obtained from the tail (WT, 499‐bp product, WT forward primer F, 5'‐AAATCCCCAGTTACTTATGGCTCC‐3', and WT reverse primer 5'‐GAGACAGTGACAGAGAGAAACCAGC‐3'; KO, 292‐bp product, KO forward primer 5'‐AGGCTATTCGGCTATGACTGGG‐3', and KO reverse primer 5'‐TGGATACTTTCTCGGCAGGAGC‐3'; Wu et al., 2011). A total of 11 male mice were provided standard chow and water ad libitum, and housed under standard laboratory conditions (20°C–26°C, 12:12‐hr light–dark cycle). At 7 weeks of age (7 weeks old; KO, *n* = 6, body weight = 20–26 g; WT, *n* = 5, body weight = 19–23 g), we cut the right sciatic nerve of all mice under anesthesia. We conducted a sham operation on the left leg of all mice under anesthesia. The animals were killed 1 week after the operation.

### 2.2 Tissue preparation

Animals were killed by cervical dislocation under anesthesia. Skeletal muscle tissue was removed and used for biochemical analysis. For these tissues, lysis buffer (50 mM HEPES [pH 7.4], 150 mM NaCl, 2 mM EDTA, 1% sodium deoxychololate, 1% NP‐40, 0.2% SDS) with phosphatase and protease inhibitors (Nacalai Tesque Inc.) was added, and the mixture was homogenized using the BEAD CRUSHER µT‐01 (TAITEC). After homogenization, the samples were centrifuged (4°C, 12,000× *g*, 15 min) and the supernatant was collected.

### 2.3 Western blotting

We used Western blotting to separate and quantify the proteins in skeletal muscle sample homogenates. First, protein content of the homogenates was measured using the bicinchoninic acid (BCA) method as per the manufacturer's instructions (Nacalai Tesque Inc.). Absorbances were measured at a wavelength of 562 nm using a microplate reader (Corona Gratong Microplate READER SH‐1000). A calibration curve was prepared using a bovine serum albumin (BSA) standard (2 mg/ml); sample concentrations were determined by comparison to the standard.

To separate the proteins, sodium dodecyl sulfate polyacrylamide gel electrophoresis (SDS‐PAGE) was conducted. Each sample was diluted with sample buffer (2 × Laemmli Sample Buffer and 10% 2‐mercaptoethanol) to a concentration of 2 mg/ml. Five microliters of each sample was loaded onto the gel. After electrophoresis, isolated proteins were transferred to a polyvinylidene fluoride membrane (PVDF; 0.3 A, 60 min). A blocking buffer containing 5% (m/v) skim milk in tris buffer containing 0.1% Tween‐20 (TBST) was used to block the PVDF membrane for 1 hr at 20°C–25°C.

Membranes were washed three times for 5 min each with TBST, incubated in the primary antibody overnight at 4°C, washed the membranes in the same frequency, and incubated the membranes in the secondary antibody (Life Technologies), diluted by 10,000‐fold with TBST, for 1 hr. The PVDF membrane was washed three times, and luminescence was detected using a chemiluminescent reagent (Immunostar Zeta or LD, Wako), and bound antibody complexes were visualized using Immunostar LD (Wako) and C‐Digit Blot Scanner (LI‐COR, Lincoln, NE). Ponceau staining was used to verify consistent loading.

We used the following primary antibodies: oxphos (ab110413, Abcam), PGC‐1α (516557, Millipore), 4–HNE (ab48506, Abcam), ubiquitin (sc‐166553, Santa Cruz), FoxO 1(2880S, Cell Signaling Technology), and p‐FoxO 1 (9461, Cell Signaling Technology), p62 (SQSTM1) (PM045, MBL), LC3 (4108, Cell Signaling Technology), Akt (9272, Cell Signaling Technology), p‐Akt (13038, Cell Signaling Technology), p70S6K (9202, Cell Signaling Technology), and p‐p70S6K (9205, Cell Signaling Technology).

### 2.4 RNA isolation and quantitative real‐time RT‐PCR

The mRNA expression was measured using real‐time RT‐PCR. To extract total RNA, the gastrocnemius muscle was homogenized on ice in Trizol reagent (Life Technologies) and then separated into organic and aqueous phases with chloroform. Samples were allowed to sit at room temperature (24°C–26°C) for 3 min and then centrifuged (4°C, 12,000× *g*, 15 min). Following precipitation with ethanol, total RNA was isolated from the aqueous phase using an RNA Basic Kit (Fast Gene). After RNA concentration was measured by spectrophotometry (Nanodrop ND1000, Thermo Scientific, altham, MA), cDNA synthesis by PrimeScriptTM RT (TAKARA, Shiga) was performed as per the manufacturer's instructions. After reverse transcription, SYBR Premix Ex taq II (Takara Bio), upstream primer, downstream primer, and RNase free water (Takara Bio) were added, and PCR was conducted. Amplification of RT‐PCR involved an initial decomposition step at 95°C for 20 s, decomposition at 95°C for 30 s, and annealing and extension at 60°C for 30 s for 40 cycles using the Thermal Cycler Dice Real‐Time System and SYBR Premix Ex taq II (Takara Bio). The mRNA expression of GAPDH was measured as the housekeeping gene. The cycle threshold (Ct) value of the target gene was standardized to the Ct value of the housekeeping gene. The expression of each target gene was calculated relative to the sham‐operated limbs. The primers sequences used in this study are shown in Table 1.

**TABLE 1:** Primer sequences used in mRNA analyses

Name	Forward (5'‐>3')	Reverse (5'‐>3')
Casp3	Tgtcatctcgctctggtacg	Tcccataaatgaccccttca
Casp6	Ttcagacgttgactggcttg	Ccagcttgtctgtctggtga
Casp7	Tttgcttactccacggttcc	Gagcatggacaccatacacg
Atg4	Tgatgggggatgggtagata	Agctttgcaggcttccacta
Atg5	Ggagagaagaggagccaggt	Tgttgcctccactgaacttg
Atg7	Aggctggctgagtcatctgt	Ggagatcttggcgttatcca

### 2.5 Statistical analysis

Data are presented as mean ± standard error of mean (*SEM*). For all measurements, a two‐way analysis of variance or *t* test was conducted. In the case of significant *F* values, comparisons were made using Tukey's post hoc test. GraphPad Prism 7 software (GraphPad, Inc.) was used for all statistical calculations, and the significance level was set to *p* < .05 for all cases.

## 3 RESULTS

### 3.1 Genotype of the renalase KO and WT mice

**FIGURE 1:**
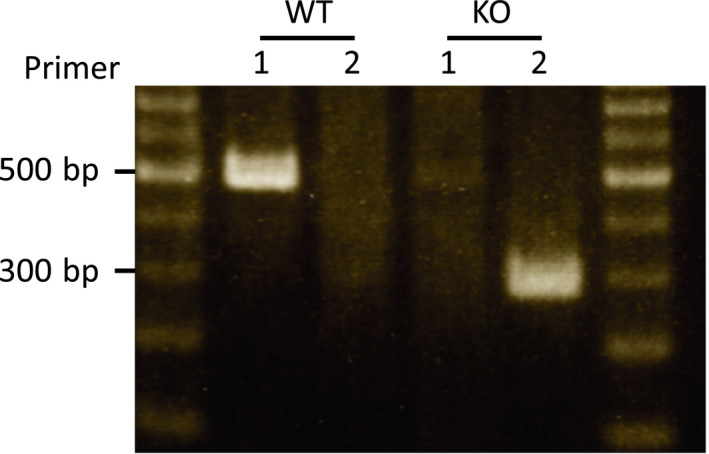
Genotype of renalase in mice. Primer 1, the array that is amplified at portion of exon 1–4; Primer 2, the array that is amplified at portion of resistance gene of neomycin. Both sides showed markers. WT, wild‐type; KO, knock out‐type

The genotypes of the mice are showed Figure 1. Renalase KO mice were developed by an exchange from exon 1–4 to the neomycin gene. Therefore, we performed PCR to confirm this gene array. We successfully detected a difference between all WT and KO mice.

### 3.2 Skeletal muscle weights

**FIGURE 2:**
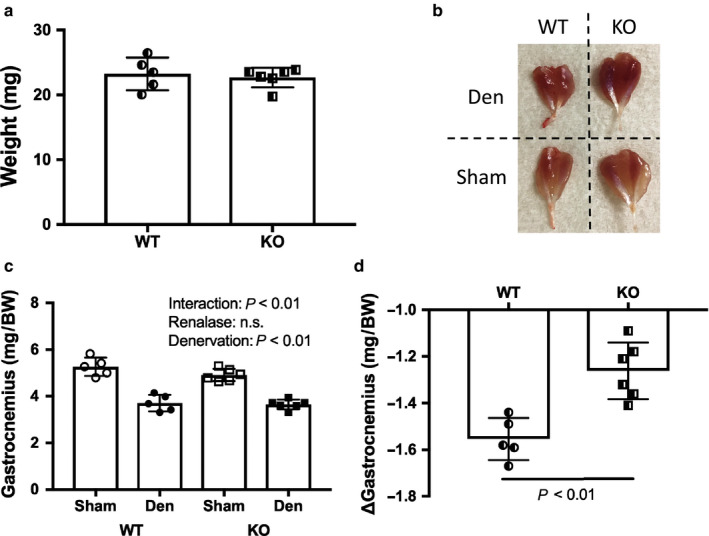
Phenotype by denervation induced muscle atrophy between WT and KO mice. (a) Body weight. (b) Image of skeletal muscles by denervation. (c) Gastrocnemius muscle weight. (d) Change in weight in gastrocnemius muscle. Data are shown as mean ± *SD*. *n* = 5 in WT group, *n* = 6 in KO group. WT, wild‐type; KO, knock out‐type; Sham, sham‐operated control; DEN, denervation.; BW, body weight

There was no difference in body weight between the WT and KO mice (Figure 2a). The weight of the gastrocnemius muscle was lower in the denervated limb than in the sham‐operated limb in the WT and KO mice (Figure 2b,c). In addition, there was a significant difference in interaction effect between the WT and KO mice. Specifically, the WT mice lost muscle at a greater rate than the KO mice (Figure 2d).

### 3.3 Mitochondria complex and PGC‐1α expression and markers of oxidative stress

**FIGURE 3:**
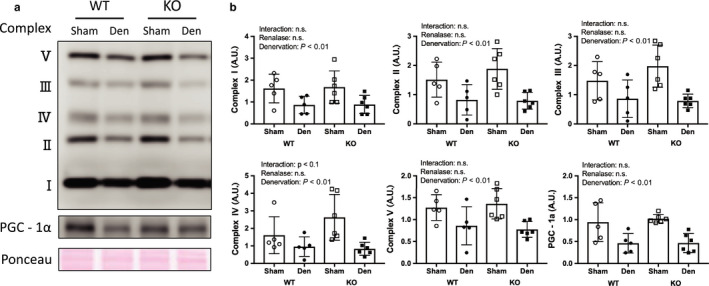
Mitochondrial complex and PGC‐1α by denervation induced muscle atrophy between WT and KO mice. (a) Image in western blotting. (b) Quantitative amount of these image in the gastrocnemius muscle by denervation. Data are shown as mean ± *SD*. *n* = 5 in WT group, *n* = 6 in KO group. WT, wild‐type; KO, knock out‐type; Sham, sham‐operated control; DEN, denervation

**FIGURE 4:**
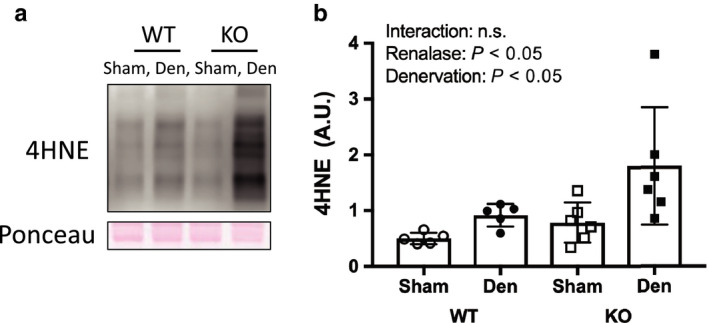
Oxidative stress by denervation induced muscle atrophy between WT and KO mice. (a) Image of 4‐hydroxy‐2‐nonenal, oxidative stress marker, in western blotting. (b) Quantitative amount of these image in the gastrocnemius muscle by denervation. Data are shown as mean ± *SD*. *n* = 5 in WT group, *n* = 6 in KO group. WT, wild‐type; KO, knock out‐type; Sham, sham‐operated control; DEN, denervation

The expression of mitochondrial respiratory chain proteins was significantly reduced in denervated muscles compared with that in sham‐operated muscles (Figure 3). In addition, PGC‐1α expression decreased significantly in denervation muscles compared with that in sham‐operated muscles (Figure 3). The oxidative stress marker 4‐HNE significantly increased in denervation muscles compared with that in sham‐operated muscles. However, there was no interaction effect between WT and KO mice (Figure 4).

### 3.4 Protein degradation pathway

**FIGURE 5:**
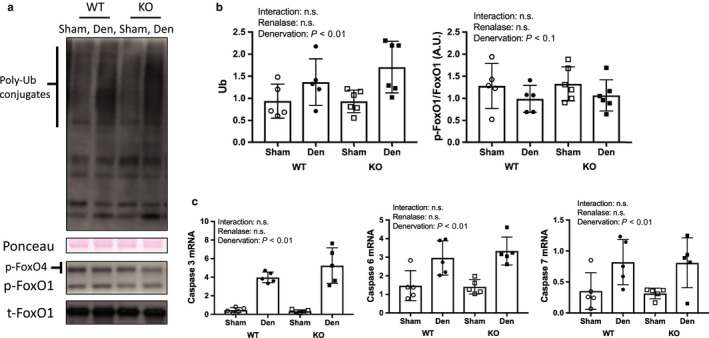
Ubiquitin proteasome and caspase pathway by denervation induced muscle atrophy between WT and KO mice. (a) Image of ubiquitin and FoxO1 in western blotting in ubiquitin and FoxO 1. (b) Quantitative amount of these image in the gastrocnemius muscle by denervation. (c) mRNA expression related to caspase pathway. Data are shown as mean ± *SD*. *n* = 5 in WT group, *n* = 6 in KO group. WT, wild‐type; KO, knock out‐type; Sham, sham‐operated control; DEN, denervation

**FIGURE 6:**
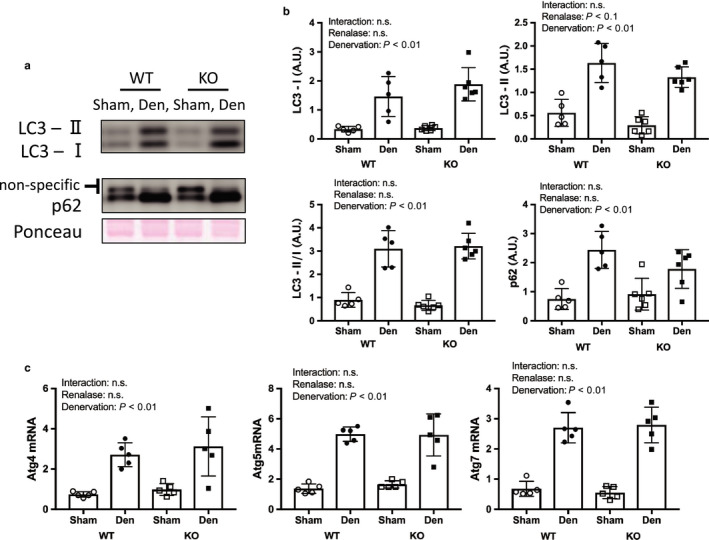
Autophagy pathway by denervation induced muscle atrophy between WT and KO mice. (a) Image of p62 and LC3 in western blotting (b) Quantitative amount of these image in the gastrocnemius muscle by denervation. (c) mRNA expression related to autophagy pathway. Data are shown as mean ± *SD*. *n* = 5 in WT group, *n* = 6 in KO group. WT, wild‐type; KO, knock out‐type; Sham, sham‐operated control; DEN, denervation

Denervation increased signaling through the protein degradation pathways (Figures 5 and 6); however, there was no difference between WT and KO mice. We observed that denervation increased the expression of ubiquitin (Figure 5a,b), mRNA of genes associated with the caspase pathway (Figure 5c), and protein and mRNA related to the autophagy pathway (Figure 6). FoxO 1 expression did not change as a result of denervation.

### 3.5 Protein synthesis pathway

**FIGURE 7:**
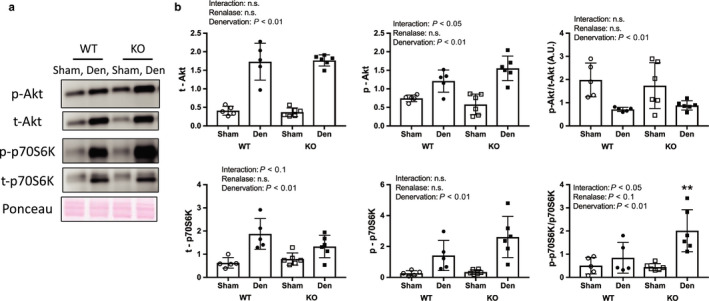
Protein synthesis pathway by denervation induced muscle atrophy between WT and KO mice. (a) Image in western blotting (b) Quantitative amount of these image in the gastrocnemius muscle by denervation. Data are shown as mean ± *SD*. *n* = 5 in WT group, *n* = 6 in KO group. WT, wild‐type; KO, knock out‐type; Sham, sham‐operated control; DEN, denervation

The expression of components of the protein synthesis pathways is shown in Figure 7. Denervation increased the protein expression of Akt and p70S6K. We observed a significant interaction for p‐Akt, despite no significant interaction for the ratio of p‐Akt/t‐Akt. Additionally, there were significant interactions for the ratio p‐p70S6K/t‐p70S6K. Moreover, the ratio of denervated muscles to sham‐operated muscles was significantly higher in the KO versus WT mice.

## 4 DISCUSSION

This study aimed to determine the effect of renalase deficiency on denervation‐induced muscle atrophy for 1 week because Castets et al. (2019) reported that protein synthesis pathway signals were increased from 1 day to 28 days on denervation model. In addition, muscle weight in denervated muscle was lighter than that in control muscle. We used denervation to induce muscle atrophy because it allowed us to compare the sham and denervated legs in a mouse model and provide an indication of the potential effects of cervical spine injury and inactivity. Our skeletal muscle mass and molecular signal findings were consistent with previous studies (Argadine et al., 2010; DePinho et al., 2014; Machida et al., 2011). Signaling through muscle degradation pathways, which include the caspase, ubiquitin–proteasome, and autophagy pathways, was increased by denervation in both renalase KO and WT mice in this study (Figures 5 and 6). However, we observed that atrophy of the gastrocnemius muscle was attenuated in KO mice compared to WT mice. This unexpected finding confirmed our hypothesis and may be explained by the two effects described in the next section.

Skeletal muscle atrophy was attenuated because of increased signaling through the protein synthesis pathway. Our results support previous studies that showed decreased muscle weight following denervation in WT mice (Machida et al., 2011). In addition, we observed increased signaling through the protein degradation pathways as a result of denervation. Unexpectedly, denervated muscles weighed more (i.e., atrophied less) in the KO mice than in the WT mice, which could have resulted from an increase in skeletal muscle protein synthesis in response to denervation‐induced muscle atrophy (DePinho et al., 2014; Machida et al., 2011). In fact, we observed greater phosphorylation of components of this pathway, such as Akt and p70S6K, in renalase KO mice than in WT mice. This suggests that renalase deficiency attenuated denervation‐induced muscle atrophy. However, it remains unclear why signaling through the protein synthesis pathway in skeletal muscle was increased following denervation. Akt is phosphorylated via the β2‐adrenal receptor (Umeki et al., 2015), and S6K is phosphorylated via the β‐adrenoceptor (Simm et al., 1998). Additionally, a previous study reported higher catecholamine concentrations in renalase KO versus WT mice (Wu et al., 2011). Thus, in renalase KO mice, the phosphorylation of Akt and S6K might be mediated by adrenaline binding to its β2‐adrenal receptor. In this study, ubiquitin–proteasome and autophagy pathway in denervated muscle did not differ between renalase KO and WT mice. The phosphorylated Akt decreases phosphorylation of FoxO (Zhao et al., 2009). In addition, Castets et al. (2019) showed that mTORC1 activation inhibits autophagy. Therefore, it is unlikely that the phosphorylated Akt relates to the result of muscle weight in this study. On the other hand, p70S6K is phosphorylated by β2‐adrenal receptor using isoproterenol, a β‐Adrenergic agonist (Pesce, Comellas, & Sznajder, 2003). Thus, it is possible that denervated muscle in KO mice received the effect of the phosphorylation of p70S6K via catecholamines. However, since we did not measure catecholamine concentrations in this study, we cannot confirm this hypothesis. We did not observe any statistical changes to the protein degradation pathway in this study. We expected to observe an increase in signaling through this pathway in renalase KO mice compared with that in WT mice because renalase plays a role in cell protection via the PMCA 4b receptor (Wang et al., 2014). We observed a significant difference in 4‐HNE (a marker of oxidative stress) between KO and WT mice, although there was no significant interaction. The overexpression of renalase reduced oxidative stress in ﻿rats that underwent unilateral ureteral obstruction (Wu et al., 2018). Thus, it is possible that oxidative stress was higher in the KO mice than in the WT mice.

Renalase phosphorylated Akt via recombinant renalase in mice whose kidneys underwent ischemia and reperfusion (Wang et al., 2014). Specifically, it was thought that renalase deficiency decreased Akt phosphorylation. However, we observed a significant difference in interaction of p‐Akt in denervated muscles between renalase KO and WT mice. Unexpectedly, we observed greater p‐p70S6K (which is downstream of Akt) in the denervated muscle of KO than in WT mice. This implies that renalase deficiency increased flux through the protein synthesis pathway, which induced the phosphorylation of p70S6K via catecholamines. In addition, Wu et al. (2011) reported that renalase deficiency led to increased circulating concentrations of catecholamines in mice. However, we were unable to elucidate the specific mechanism underlying this effect in this study because of analysis of skeletal muscle sample only.

## 5 CONCLUSIONS

Renalase deficiency attenuated denervation‐induced muscle atrophy. The mechanism underlying this effect might be related to catecholamines, because signaling through the protein synthesis pathway was increased following denervation in renalase KO mice compared with that in WT mice, despite showing no change in protein degradation. In future studies, protein synthesis and degradation pathways on denervation‐induced muscle atrophy in conditional renalase‐deficient mice, that is, skeletal muscle specific KO renalase mice.

## CONFLICT OF INTEREST

None declared.
